# Insecticide resistance status, frequency of L1014F *Kdr* and G119S *Ace-1* mutations, and expression of detoxification enzymes in *Anopheles gambiae* (*s.l.*) in two regions of northern Benin in preparation for indoor residual spraying

**DOI:** 10.1186/s13071-018-3180-2

**Published:** 2018-12-04

**Authors:** Albert Sourou Salako, Idelphonse Ahogni, Rock Aïkpon, Aboubakar Sidick, Fortune Dagnon, Arthur Sovi, André Aimé Sominahouin, Fiacre Agossa, Laurent Iyikirenga, Martin C. Akogbeto

**Affiliations:** 1 Centre de Recherche entomologique de Cotonou (CREC), Cotonou, Benin; 2 Faculté des Sciences et Techniques de l’Université d’Abomey-Calavi, Abomey-Calavi, Benin; 3 Technologies, Ingénierie et Mathématiques, Université Nationale des Sciences, Abomey, Bénin; 4 US Agency for International Development, US President’s Malaria Initiative, Cotonou, Benin; 5 PMI VectorLink project, Abt associates, Bamako, Mali; 6 Faculté des Sciences Humaines et Sociales de l’Université d’Abomey-Calavi, Abomey-Calavi, Benin; 7 PMI VectorLink project, Abt associates, Cotonou, Benin

**Keywords:** Resistance, *Anopheles gambiae* (*s.l.*), IRS, Benin

## Abstract

**Background:**

This study aims to provide baseline data on the resistance status to insecticides, the frequency of mechanisms involved and the impact of the association with the synergist piperonyl butoxide (PBO) on resistant *Anopheles gambiae* (*s.l.*) populations in two regions of northern Benin, prior to an indoor residual spraying campaign and introduction of next generation long-lasting insecticidal nets (LLINs) incorporating PBO.

**Methods:**

Adult *Anopheles gambiae* (*s.l.*) originating from larvae collected in two study regions (Alibori within the Kandi-Gogounou-Segbana districts and Donga within the Djougou-Copargo-Ouake districts) were tested with impregnated papers (bendiocarb 0.1%, pirimiphos-methyl 0.25%, permethrin 0.75% and deltamethrin 0.05%). The synergist PBO was used to check for the involvement of detoxification enzymes in pyrethroid resistant populations. Molecular analyses were performed for the identification of species within the *Anopheles gambiae* (*s.l.*) complex and *kdr* L1014F and G119S *Ace-1* mutations. Biochemical assays assessed the activity of detoxification enzymes.

**Results:**

*Anopheles gambiae* (*s.l.*) was resistant to pyrethroids, with a mortality range of 25–83% with deltamethrin and 6–55% with permethrin. A significant increase in mortality was observed after pre-exposure to PBO for both deltamethrin (63–99%) and permethrin (56–99%). With bendiocarb, *An. gambiae* (*s.l.*) were susceptible in Kandi (99% mortality), with possible resistance (92–95%) recorded in Djougou, Copargo, Gogounou, Ouake and Segbana. All study populations were fully susceptible to pirimiphos-methyl. The frequencies of resistant mutations varied according to species and sites: 0.67–0.88 for L1014F *kdr* and 0–0.06 for G119S *Ace-1*. Three study locations (Djougou, Gogounou and Kandi) showed high oxidase activity and four sites (Djougou, Ouake, Copargo and Kandi) showed elevated esterase activity.

**Conclusions:**

This study confirms resistance to pyrethroids and suggests emerging bendiocarb resistance in *An. gambiae* (*s.l.*) populations in northern Benin. However, recovery of susceptibility to pyrethroids after PBO exposure, and susceptibility to organophosphates in the *An. gambiae* (*s.l.*) populations indicate that next generation LLINs incorporating PBO synergist combined with an indoor residual spraying (IRS) campaign with organophosphate insecticides may be regarded as alternative control tools.

## Background

Vector control is an essential component in malaria prevention strategies [1]. In Africa, it relies primarily on two effective and complementary tools: long-lasting insecticidal nets (LLINs) and indoor residual spraying (IRS) [2–4]. Several studies have demonstrated the effectiveness of both tools in reducing the incidence of malaria [5, 6] morbidity and mortality in Africa [7–10]. In Benin, malaria vector control relies mainly on the mass distribution of LLINs, and on IRS operations. From 2008 to 2015, IRS with bendiocarb (a carbamate) in southern Benin and with pirimiphos-methyl (an organophosphate) in the northern region, showed a significant reduction in malaria transmission [11, 12]. Although LLINs and IRS have been shown to be effective, they have performed below expectations in some settings, including several locations in Benin [13–15]. One of the reasons is the emergence and expansion of resistance of *Anopheles* vectors to insecticides, especially pyrethroids [16–23] and more recently bendiocarb [24–26]. The main insecticide resistance mechanisms involve an increase in the activity of detoxification enzymes (oxidases, esterases and glutathione-S-transferases) [18, 19, 27, 28] and the *kdr* L1014F and G119S *Ace-1* target site mutations frequently found in *An. gambiae* (*s.l.*) populations [16, 29–31]. Studies suggest that the use of the same classes of insecticides in public health as well as in agriculture, especially in cotton cultivation, may have led to the increase in the allelic frequencies of the *kdr* L1014F and G119S *Ace-1* in Benin [22, 23, 25, 26, 32, 33]. In Benin, the IRS program implemented in 2017 targeted all houses in the regions of Alibori and Donga with pirimiphos-methyl. In the same year, the Benin national malaria control program (NMCP), supported by USAID, Global Fund and the WHO, undertook large-scale distribution of Yorkool LLINs impregnated with deltamethrin. In preparation for the implementation of these two control campaigns, the present study was initiated to collect data on the resistance of vectors to insecticides in the two targeted regions. These baseline data inform selection of insecticide candidates for IRS and help to define strategies for effective insecticide resistance management in the study area.

## Methods

### Study regions and mosquito sampling sites

**Fig. 1:**
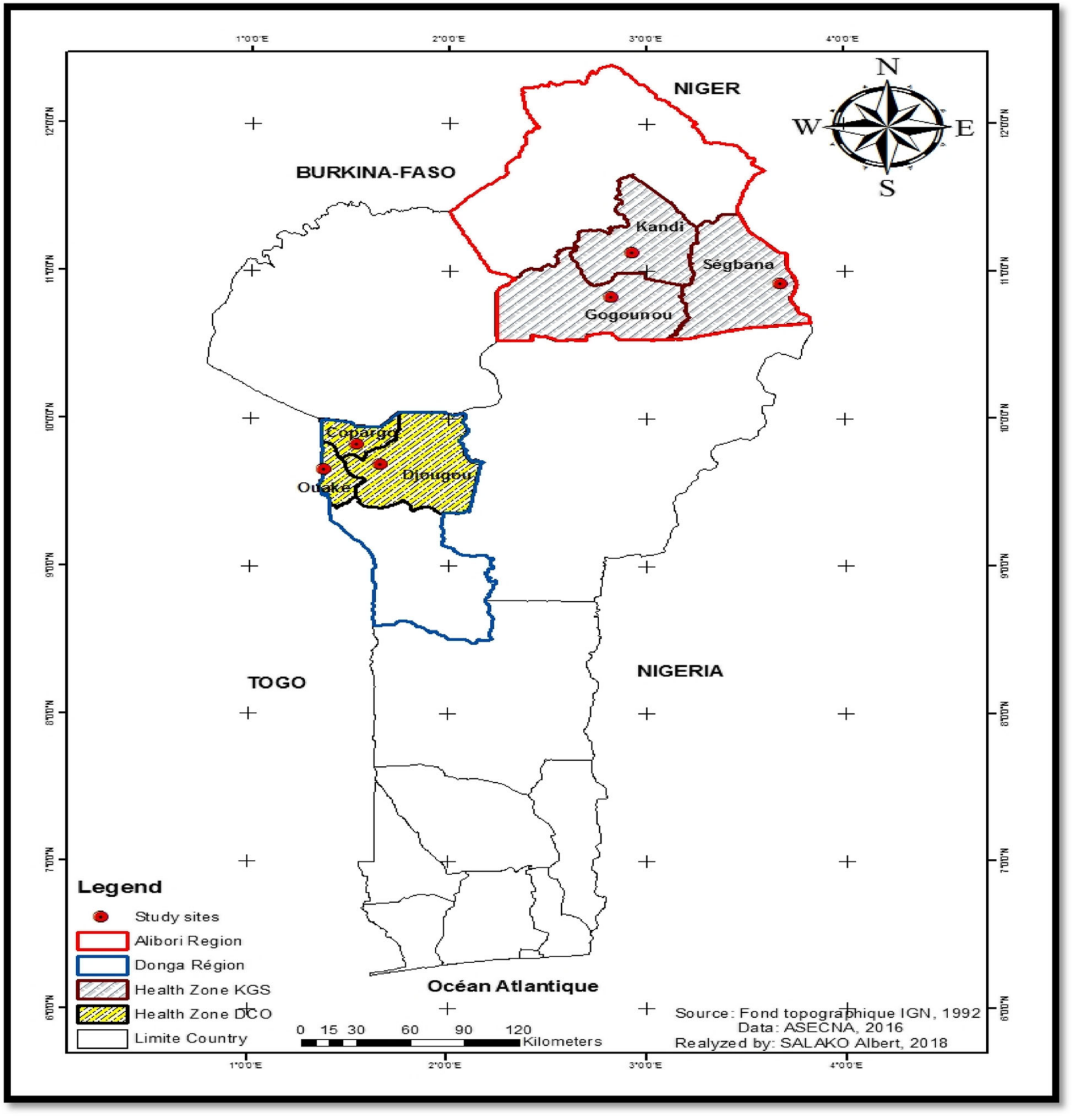
Study area showing the six surveyed districts

The study was conducted during the rainy season (June to October 2016) in six districts of northern Benin. These six districts are grouped into two healthcare facility’s catchment areas: the Kandi-Gogounou-Segbana health zone (KGS) located in the Alibori region and the Djougou-Copargo-Ouake health zone (DCO) in the Donga region. The general census of the population and housing carried out in May 2013 revealed estimated populations of 867,463 and 543,130 inhabitants in Alibori and Donga, respectively [34]. These two regions are located in a dry savanna area and in a dry and wet savanna area, respectively (Fig. 1). The Alibori region is crossed by several rivers and water dams and the soil is sandy. In the Donga region, the soil is clay.

### Study areas

#### Kandi-Gogounou-Segbana (KGS) health zone

This area covers approximately 12,943 km^2^ and is the largest health zone in Benin. It is composed of three districts: Kandi (11°07' to 29.32'N, 2°56' to 9.57'E), Gogounou (10°33' to 10°57'N, 2°15' to 3°15'E) and Segbana (10°32' to 11°23'N, 3°08' to 3°50'E) (Fig. 1). It is recognized as the cotton cultivation area of northern Benin, with large quantities of insecticides used to control cotton pests [35]. Its climate is Sudanese with two seasons a year: a single rainy season from May to October and a dry season from November to April. The mean temperature and relative humidity are about 28 °C and 70%, respectively. Rainfall varies between 700–1200 mm with heavy rainfall recorded between July and September [36].

#### Djougou-Copargo-Ouake (DCO) health zone

This health zone covers an area of ​​5465 km^2^ and is composed of three districts: Djougou (09°42' to 10°1'N, 01°40' to 55°4'E), Copargo (09°50' to 19°3'N, 01°32' to 39°5”E) and Ouake (09°40' to 45.3'N, 01°22' to 51°7'E) (Fig. 1). In this zone, Djougou is the district where cotton cultivation is highly developed with a high use of insecticide [35]. It has a Sudano-Guinean climate with two seasons. The rainy season extends over 6 months (from mid-April to mid-October). The average rainfall is between 1200–1300 mm. The mean temperature is around 27 °C. The main crops are yam, cereal and cotton [37].

### Mosquito collections

Larvae of *Anopheles* mosquitoes were collected in breeding sites using the dipping technique. Larvae and pupae were collected from various breeding sites (e.g. rain water collection, irrigation channels, river beds, wells, etc.), so that the mosquitoes tested were fully representative of the vector population in the area.

Insufficient numbers of larvae were collected at Segbana and Ouake to perform all of the susceptibility bioassay tests. Species were identified using a morphological key [38].

### Insecticide susceptibility tests

Susceptibility tests were performed using the WHO tube bioassay test [39]. The following insecticides and synergist were tested: deltamethrin (0.05%), permethrin (0.75%), deltamethrin (0.05%) + PBO (4%), permethrin (0.75%) + PBO (4%), bendiocarb (0.1%), and pirimiphos-methyl (0.25%).

The PBO synergist was used to evaluate the involvement of detoxification enzymes (oxidases and esterases) [40] in the phenotypic resistance of the populations of *An. gambiae* (*s.l.*) of each district.

About 100 adult female mosquitoes were exposed to each insecticide, tested in 4 replicates each of *c.*25 mosquitoes. In addition, 50 mosquitoes served as controls in 2 replicates of *c.*25 mosquitoes. Knockdown was recorded at 10, 15, 20, 30, 40, 50 and 60 min. All tests were conducted at 25 °C and 80% humidity.

Mortality after 24 h was determined and interpreted according to the WHO protocol [39]. At the end of the tests, live and dead specimens from each district were used for species identification and determination of resistance mechanisms (*kdr* L1014F, *kdr* L1014S and G119S *Ace-1*) using PCR methods.

According to the site, 72–270 individuals randomly selected from live and dead mosquitoes from susceptibility tests were analyzed according to the protocol of Santolamazza et al. [41] to determine species within the *An. gambiae* (*s.l.*) complex. The same mosquitoes were genotyped for the *kdr* L1014F, *kdr* L1014S and *G119S Ace-1* mutations, according to the protocols of Martinez-Torres et al. [29], Ranson et al. [30] and Weill et al. [42], respectively.

### Biochemical analyses

Thirty females of *An. gambiae* (*s.l.*) from each district of the KGS and DCO health zones, aged 2–5 days and which were not previously used for any insecticidal test, were used for biochemical analyses. Biochemical assays were performed to compare the level of activities of mixed function oxidases (MFOs), non-specific esterases (α and β-esterases) and glutathione S-transferases (GSTs) [43] of the different field mosquito populations to the Kisumu susceptible strain. All mosquitoes were tested according to the protocol described by Hemingway et al. [44]. Oxidase activity was assessed with the heme-peroxidase test which allowed detection of the increase in the quantity of heme. Alpha-Naphthol acetate (αNaph) (Sigma N-1000, Saint Louis, Missouri, USA) and Beta-Naphthol acetate (βNaph) (Sigma N-185507) were used to evaluate the non-specific esterase activity. GST activity was determined by measuring in time the formation of the Glutathione-S-CDNB at 340 nm after a catalysis reaction between the 1-chloro-2,4-dinitrobenzene (CDNB) and reduced glutathione (GSH).

### Data analysis

Any mosquito population with a mortality rate between 98–100% was considered susceptible. When mortality was between 90–97%, the population was suspected of resistance. Below a 90% mortality rate, the population was considered resistant. The mortality rates of populations of *An. gambiae* (*s.l.*) were compared using a Chi-square test of comparison of proportions. The allelic frequencies of *kdr* L1014F and G119S *Ace-1* were calculated as follows: F(R) = [2n.RR+ n.RS]/[2(n.RR+ n.RS+ n.SS)] [45] (n. is the number of mosquitoes of a given genotype), to assess their variability across populations. A linear regression with variance analysis was used to assess the variation of enzymatic activity in each locality. Mann-Whitney U-test was used to compare enzyme activity between field- and laboratory-susceptible mosquitoes (Kisumu). Statistical analyses were performed with software R 3.3.2 [46].

## Results

### Mortality rates of *An. gambiae* (*s.l.*)

**Fig. 2:**
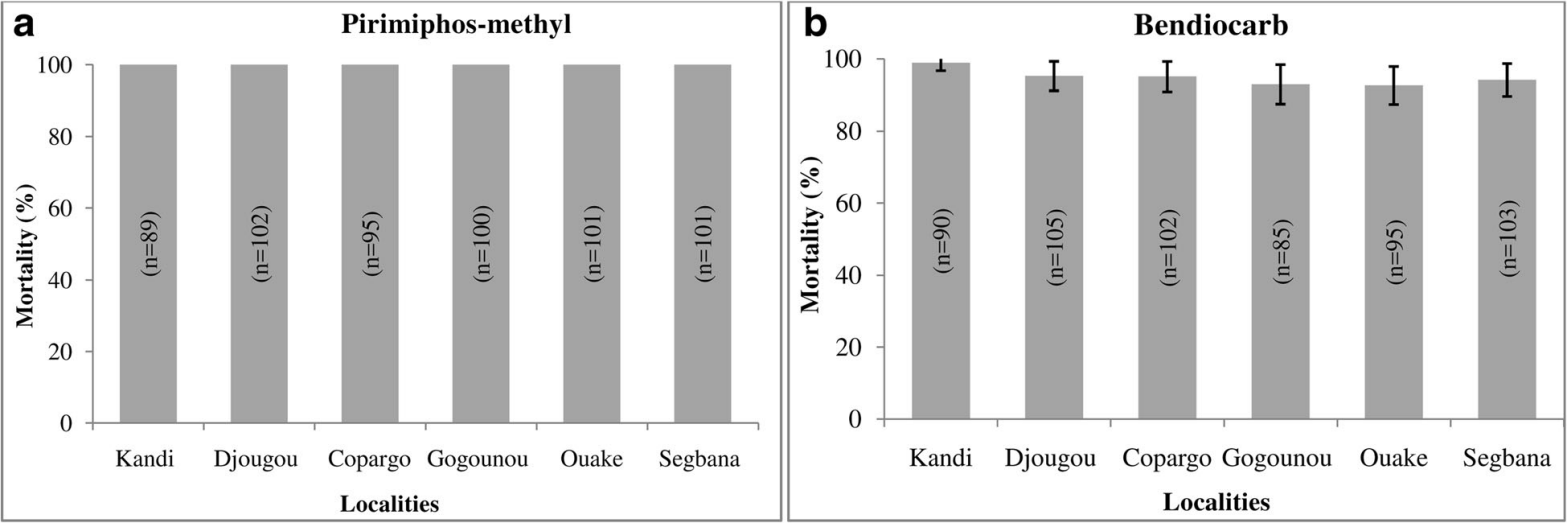
Mortalities observed 24 hours after mosquito exposure to pirimiphos-methyl (**a**) and bendiocarb (**b**)

With pirimiphos-methyl, the mortality rates observed in all tested populations were 100%, thus showing full susceptibility (Fig. 2a).

The mortality rate observed with bendiocarb in Kandi was 98.88% (Fig. 2b), which shows susceptibility of this mosquito population. In the other five districts, possible resistance was observed with mortality rates varying from 92.63% in Ouake to 95.23% in Djougou (Fig. 2b).

**Fig. 3:**
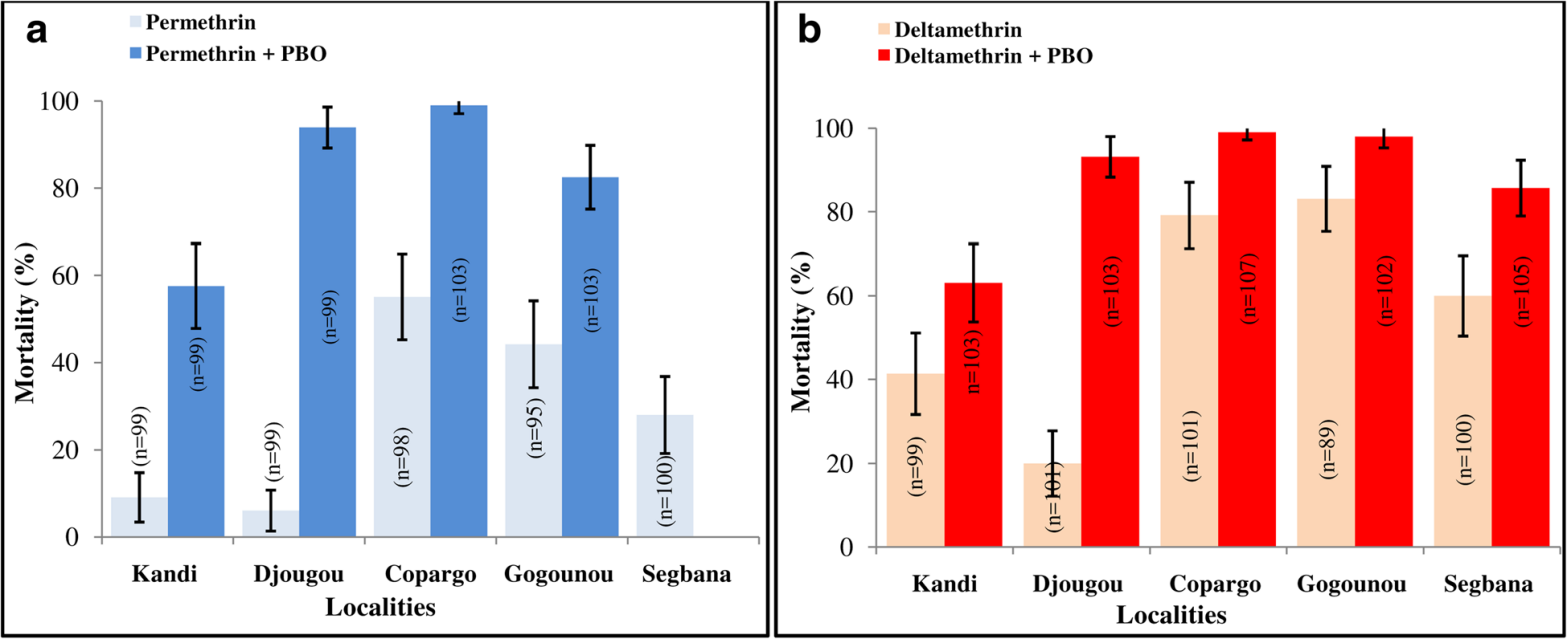
Mortality rates of *An. gambiae* (*s.l*.) with permethrin alone and permethrin + PBO (**a**), deltamethrin alone and deltamethrin + PBO (**b**) in the surveyed districts, 24 hours after exposure

All populations of *An. gambiae* (*s.l.*) from the surveyed districts, were found to be resistant to permethrin with mortality rates ranging from 6.06% in Djougou to 55.1% in Copargo) (Fig. 3a). In Djougou, Kandi, Gogounou and Copargo, the mortality rates increased from 6.06, 9.09, 44.21 and 55.1%, respectively, with permethrin alone to 93.93% (*χ*^2^ = 149.41, *df* = 1, *P* < 0.0001), 57.57% (*χ*^2^ = 50.205, *df* = 1, *P* < 0.0001), 82.52% (*χ*^2^ = 53.269, *df* = 1, *P* < 0.0001) and 99.02% (*χ*^2^ = 53.269, *df* = 1, *P* < 0.0001) with permethrin + PBO (Fig. 3a).

All populations tested were resistant to deltamethrin with mortality rates ranging from 25.27% (Djougou) to 83.14% (Gogounou) (Fig. 3b). With pre-exposure to PBO, an increase in susceptibility to deltamethrin was noted. At Copargo, Gogounou, Djougou, Kandi and Segbana, mortality rates increased from 79.2, 83.14, 25.27, 41.41 and 60%, respectively, with deltamethrin alone to 99.06% (*χ*^2^ = 19.613, *df* = 1, *P* = 0.0009), 98.03% (*χ*^2^ = 11.23, *df* = 1, *P* = 0.0008), 93.20% (*χ*^2^ = 109.04, *df* = 1, *P* < 0.0001), 63.10% (*χ*^2^ = 8.675, *df* = 1, *P* = 0.0322) and 85.71% (*χ*^2^ = 15.967, *df* = 1, *P* = 0.0064) with deltamethrin + PBO (Fig. 3b).

### Distribution of sibling species of the *Anopheles gambiae* complex by site and in dead and live mosquitoes

Out of the 1163 specimens of *An. gambiae* (*s.l.*) analyzed by PCR in the two investigated health zones, 55.46% were *An. gambiae* and 44.54% *An. coluzzii*. Overall, *An. coluzzii* was predominant in Kandi and Ségbana with a mean of 61.6% in the KGS health zone as compared to *An. gambiae* (38.4%). In the DCO health zone, *An. gambiae* was in majority in all three districts (Djougou, Copargo and Ouaké) with a mean of 68.92% compared to *An. coluzzii* (31.08%) (Table 1).

**Table 1:** Distribution of *An. coluzzii* and *An. gambiae* in the KGS and DCO health zones

Heath zone	District	Total no. tested	*An. coluzzii*		*An. gambiae*		*χ*^2^-value	*df*	*P*-value^a^
			*n*	%	*n*	%			
KGS	Kandi	266	180	67.67	86	32.33	65.03	1	<0.001
	Gogounou	175	91	52	84	48	0.4114	1	0.5212
	Segbana	72	45	62.5	27	37.5	8.027	1	0.004
	Total (KGS)	513	316	61.60	197	38.40	54.28	1	<0.001
DCO	Djougou	269	101	37.55	168	62.45	32.38	1	<0.001
	Copargo	270	51	18.89	219	81.11	206.59	1	<0.001
	Ouake	111	50	45.05	61	54.95	1.801	1	0.179
	Total (DCO)	650	202	31.08	448	68.92	184.69	1	<0.001
	Grand total	1163	518	44.54	645	55.46	27.302	1	<0.001

^a^*% An. coluzzii vs* % *An. gambiae*

Overall, in Kandi, Gogounou and Segbana, mortality occurred mostly in *An. coluzzii* as compared to *An. gambiae* (*χ*^2^ = 13.357, *df* = 1, *P* = 0.0003 and *χ*^2^ = 13.837, *df* = 1, *P* = 0.0002, for bendiocarb and deltamethrin, respectively) in the KGS health zone (Table 2). By contrast, in the DCO health zone, mortality occurred similarly in *An. gambiae* and *An. coluzzii* (*χ*^2^ = 1.456, *df* = 1, *P* = 0.227 for bendiocarb; *χ*^2^ = 0.482, *df* = 1, *P* = 0.487 for permethrin; and *χ*^2^ = 0.0359, *df* = 1, *P* = 0.849 for deltamethrin) (Table 2).

**Table 2:** Number of *An. coluzzii* and *An. gambiae* in dead and live mosquitoes from the KGS and DCO health zones

Health Zone	District	Total no. tested	Bendiocarb				Permethrin				Deltamethrin				Pirimiphos-methyl			
			*An. coluzzii*		*An. gambiae*		*An. coluzzii*		*An. gambiae*		*An. coluzzii*		*An. gambiae*		*An. coluzzii*		*An. gambiae*	
			Dead (*n*)	Live (*n*)	Dead (*n*)	Live (*n*)	Dead (*n*)	Live (*n*)	Dead (*n*)	Live (*n*)	Dead (*n*)	Live (*n*)	Dead (*n*)	Live (*n*)	Dead (*n*)	Live (*n*)	Dead (*n*)	Live (*n*)
KGS	Kandi	266	53	0	14	1	8	58	1	32	34	27	7	31	nt	nt	nt	nt
	Gogounou	175	28	2	24	4	16	15	16	12	24	6	20	8	nt	nt	nt	nt
	Segbana	72	15	0	3	6	5	9	1	9	15	1	2	6	nt	nt	nt	nt
	Total (KGS)	513	96	2	41	11	29	82	18	53	73	34	29	45	nt	nt	nt	nt
	Proportion (%)		97.9	2.1	78.9	21.1	26.1	73.9	25.4	74.6	68.2	31.8	39.2	60.8	nt	nt	nt	nt
DCO	Djougou	269	35	0	53	5	4	28	2	54	13	21	8	46	nt	nt	nt	nt
	Copargo	270	18	1	74	4	14	4	33	31	13	1	59	18	nt	nt	nt	nt
	Ouake	111	41	2	47	5	nt	nt	nt	nt	nt	nt	nt	nt	7	0	9	0
	Total (DCO)	650	94	3	174	14	18	32	35	85	26	22	67	64	7	0	9	0
	Proportion (%)		96.9	3.1	92.6	7.4	36.0	64.0	29.2	70.8	54.2	45-8	51.0	49.0	100	0	100	0

*Abbreviation*: *nt*, no mosquitoes tested by PCR to identify species

### Distribution of L1014F *kdr* and G119S *Ace-1* mutations in *An. gambiae* and *An. coluzzii*

Tables 3 and 4 show the distribution of the frequency of the *kdr* L1014F and G119S *Ace-1* mutations in *An. gambiae* and *An. coluzzii* in the six surveyed districts. Overall, the mean frequency of the *kdr* L1014F gene at all districts was 0.77. These frequencies were higher in *An. gambiae* than in *An. coluzzii* at all sites with a significant difference between the frequencies of the two sibling species at Kandi and Gogounou (Table 3). The *kdr L1014S* resistant allele was not detected in our samples.

**Table 3:** Frequencies of the *kdr* L1014F mutation observed in *An. gambiae* and *An. coluzzii*

District	Species	No. tested	Genotype			Freq. 1014F	*χ*^2^-value	*df*	*P*-value
			1014F/F	1014L/F	1014L/L				
Kandi	*An. gambiae*	86	57	19	10	0.773	7.043	1	0.0079
	*An. coluzzii*	180	86	64	30	0.656			
Gogounou	*An. gambiae*	84	66	15	3	0.875	5.924	1	0.0149
	*An. coluzzii*	91	57	26	8	0.769			
Segbana	*An. gambiae*	27	15	9	3	0.722	0.260	1	0.609
	*An. coluzzii*	45	22	16	7	0.667			
Djougou	*An. gambiae*	168	114	41	13	0.801	2.534	1	0.111
	*An. coluzzii*	101	63	23	15	0.738			
Copargo	*An. gambiae*	219	158	52	9	0.840	0.440	1	0.506
	*An. coluzzii*	51	39	11	1	0.873			
Ouake	*An. gambiae*	61	44	11	6	0.811	3.770	1	0.0521
	*An. coluzzii*	50	27	15	8	0.690			
Total	*An. gambiae*	645	454	147	44	0.818	32.599	1	<0.0001
	*An. coluzzii*	518	294	155	69	0.717			

*Abbreviation*: *Freq*., frequency

**Table 4:** Frequencies of the *Ace1* G119S mutation observed in *An. gambiae* and *An. coluzzii*

District	Species	No. tested	Genotype			Freq. 119S	*χ*^2^-value	*df*	*P*-value
			119S/S	119G/S	119G/G				
Kandi	*An.gambiae*	86	0	4	82	0.0233	0.179	1	0.671
	*An. coluzzii*	180	0	5	175	0.0139			
Gogounou	*An.gambiae*	84	0	6	78	0.0357	1.412	1	0.234
	*An. coluzzii*	91	0	2	89	0.011			
Segbana	*An.gambiae*	27	0	3	24	0.0556	2.746	1	0.0974
	*An. coluzzii*	45	0	0	45	0			
Djougou	*An.gambiae*	168	0	10	158	0.0298	0.178	1	0.672
	*An. coluzzii*	101	0	4	97	0.0198			
Copargo	*An.gambiae*	219	0	10	209	0.0228	1.282	1	0.257
	*An. coluzzii*	51	0	0	51	0			
Ouake	*An.gambiae*	61	0	2	59	0.0164	<0.0001	1	1
	*An. coluzzii*	50	0	1	49	0.01			
Total	*An.gambiae*	645	0	35	610	0.0271	6.252	1	0.0124
	*An. coluzzii*	518	0	12	506	0.0116			

*Abbreviation*: *Freq*., frequency

The G119S *Ace-1* mutation was identified in all districts at very low frequency (between 1–6%) (Table 4). It varied from 2 to 6% in *An. gambiae* and from 0 to 2% in *An. coluzzii* (Table 4) with a significant difference between both species when all sites were combined.

### Expression of oxidases, esterases and GSTs in *An. gambiae* (*s.l.*)

**Fig. 4:**
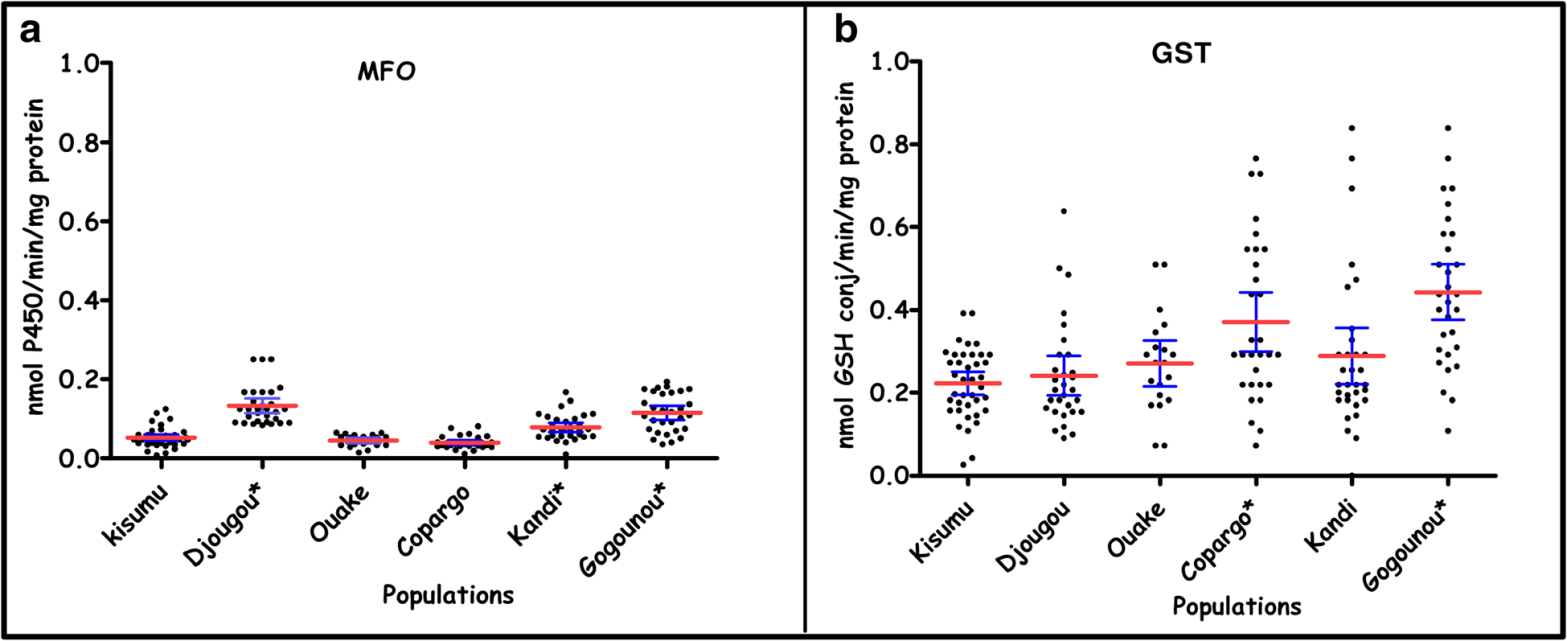
Mono-oxygenase (**a**) and glutathione-S-transferase (**b**) activities in field populations of *Anopheles gambiae* (*s.l.*). *Population with a significantly higher enzyme activity as compared to the Kisumu reference susceptible strain. The red horizontal lines indicates the mean level of enzymatic activity. *Abbreviations*: MFO, Mixed function oxidases; GST, glutathione S-transferase

**Fig. 5:**
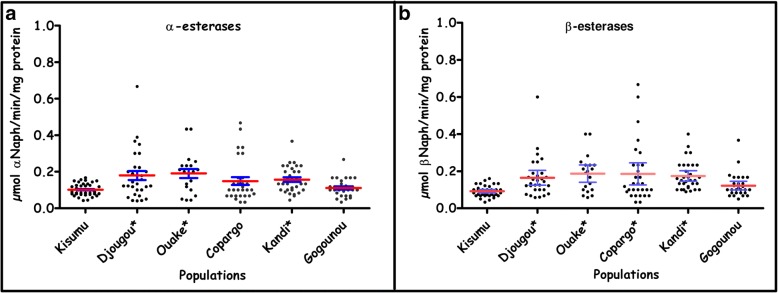
α-esterase and β-esterase activities in field populations of *Anopheles gambiae* (*s.l.*). *Population with a significantly higher enzyme activity as compared to the Kisumu reference susceptible strain. The red horizontal lines indicates the mean level of enzymatic activity

Figures 4 and 5 display the mean levels of enzymatic activities in field mosquito populations and the Kisumu reference susceptible strain. In all investigated districts, at least one class of detoxification enzyme revealed elevated activity relative to the Kisumu strain. Oxidase activity was significantly elevated in the districts of Djougou (Mann-Whitney U-test, *U* = 48.50, *P* < 0.0001), Gogounou (*U* = 149.5, *P* < 0.0001) and Kandi (*U* = 280.5, *P* < 0.0001) compared to the Kisumu strain (Fig. 4a). The highest glutathione-S-transferase (GST) activities were observed in the Copargo and Gogounou populations with a significant difference compared to the Kisumu strain (*U* = 312, *P* = 0.0009 and *U* = 151.1, *P* < 0.0001, respectively) (Fig. 4b).

The activity of α esterases was higher in the populations of Djougou (*U* = 369, *P* = 0.009), Ouake (*U* = 190, *P* = 0.0014) and Kandi (*U* = 322, *P* = 0.0005) compared to the Kisumu strain (Fig. 5a). Significantly elevated β esterase activities were observed in Djougou (*U* = 265.5, *P* = 0.0001), Ouake (*U* = 157, *P* = 0.0002), Copargo (*U* = 357, *P* = 0.0055) and Kandi (*U* = 144, *P* < 0.0001) compared to the Kisumu strain (Fig. 5b).

## Discussion

Monitoring is an integral part of any resistance management strategy which allows informed decisions about the choice of insecticides [47]. The present study shows confirmed resistance of malaria vectors to deltamethrin and permethrin (pyrethroids), increased susceptibility to pyrethroids through the use of PBO, decreased susceptibility to bendiocarb in some districts and full susceptibility to pirimiphos-methyl.

The predominance of *An. coluzzii* in Alibori could be due to the presence of rivers and water dams that create numerous permanent and semi-permanent larval habitats conducive to the emergence of this mosquito species. In addition, the sandy soil of the region may cause a fast infiltration of water after rainfall, which would favor the formation of only very few temporary breeding sites. By contrast, the clay soil in Donga retains water after rainfall and, as a result, several temporary larval habitats could be formed, thus allowing the development of *An. gambiae* which was in majority in this region.

In the present study, levels of resistance to permethrin and deltamethrin observed after recording the 24-h mortality rates varied between districts. Pyrethroid resistance in *An. gambiae* (*s.l.*) observed in the present study confirms findings of previous studies carried out in Benin [22, 23, 25]. The lowest mortality rates to pyrethroids were observed in the districts of Kandi, Djougou and Segbana. This could be due to the strong selection pressure exerted by the large-scale cotton production [35]. The wide distribution and high level of malaria vectors’ resistance to pyrethroids might be due to the expansion of agriculture [48, 49] and the mass use of pyrethroid-treated mosquito nets, distributed at the national level over past years [50, 51]. As mortality occurred mostly in the predominant species (*An. coluzzii*) in the KGS health zone but was similar in each species in the DCO health zone, no conclusion can be drawn on a higher susceptibility to insecticides between the species.

Overall, *An. gambiae* (*s.l.*) was resistant to pyrethroids and displayed high frequencies of the *kdr* L1014F mutation in all surveyed districts. The *kdr* L1014F frequency was higher in *An. gambiae* than in *An. coluzzii* in most localities, which confirms the recent findings of Gnanguenon et al. [26] and Yahouédo et al. [52] in some sites located on the north-south axis of Benin. Several previous studies have also shown that frequencies of *kdr* L1014F are higher in *An. gambiae* in west and central Africa compared to *An. coluzzii* [41, 53], except for some urban and peri-urban coastal areas [54]. In addition to *kdr* L1014F that could compromise the effectiveness of vector control tools such as LLINs and IRS [55], the involvement of mono-oxygenases in pyrethroids resistance in our study sites has also been noted since the level of vector resistance to deltamethrin and permethrin was significantly reduced by the use of PBO. These oxidases are involved in the detoxification of pyrethroids in *Anopheles gambiae* (*s.l.*) [56, 57]. Moreover, our biochemical data have revealed their overexpression in *An. gambiae* (*s.l.*) in Djougou, Gogounou and Kandi. This result is similar to that obtained by Djouaka et al. [27] in natural populations of *An. funestus* (*s.l.*) in Pahou and confirms the works of Djègbè et al. [22] and Aizoun et al. [24] at Kandi and Malanville, respectively, two sites of northern Benin near our study area. The simultaneous presence of *kdr* L1014F and elevated oxidase activity could confer higher resistance in mosquitoes. In these conditions, the use of PBO LLINs (Permanet 3.0 nets and Olyset Plus mosquito nets) associated with a pirimiphos-methyl-based IRS could be implemented for effective malaria vector control in Alibori and Donga, two regions selected to receive both interventions. Indeed, a recent study carried out by Protopopoff et al. [58] in Tanzania reported a better performance of the strategy combining PBO LLINs and IRS with pirimiphos-methyl on malaria transmission as compared to standard LLINs, in pyrethroids resistance areas. The high activity of glutathione-S-transferases in the wild populations of *An. gambiae* (*s.l.*) in the districts of Copargo and Gogounou could play a minor role in the resistance to pyrethroids due to the oxidative stress [59]. Other studies attribute this overexpression of GSTs to the resistance of *An. gambiae* (*s.l.*) to DDT [19]. In such case, in the presence of *kdr* L1014F, GSTs could increase phenotypic resistance to pyrethroids and DDT and broaden the spectrum of resistance to independent compounds [60].

The possible resistance to bendiocarb (carbamate) and the low frequencies of the G119S *Ace-1* observed in our study sites was also previously reported by Djenontin et al. [61]. This start of resistance to bendiocarb and the observed presence of some heterozygous (RS) individual mosquitoes for the G119S *Ace-1R* mutation is worrying given several studies have shown that this insecticide represents a potential alternative to pyrethroids for the management of resistance [62, 63]. The highest frequencies of G119S *Ace-1R* were found in *An. gambiae* (2–6%) and the lowest in *An. coluzzii* (0–2%). These results corroborate those obtained by Aikpon et al. [25] and Gnanguenon et al. [26] in the Atacora and Kandi districts of Benin, respectively. However, even though the G119S *Ace-1* mutation is often incriminated in vectors resistant to carbamates and organophosphates, it does not fully explain the observed possible resistance to bendiocarb, because some susceptible homozygous individuals survive after exposure to this carbamate [54]. Susceptibility of *An. gambiae* (*s.l.*) to pirimiphos-methyl observed in our study area confirms the findings of Asidi et al. [64] which showed that the presence of G119S *Ace-1* does not confer a systematic resistance to organophosphates.

## Conclusions

With high pyrethroid resistance, overexpression of some metabolic enzymes (MFO, GST) and the high *kdr* L1014F allelic frequencies observed in *An. gambiae* (*s.l.*) in the KGS and DCO health zones, IRS with pirimiphos-methyl - for which full susceptibility was detected - is recommended for control. Furthermore, the increased susceptibility level of vectors to pyrethroids after pre-exposure to PBO suggests that implementation of PBO-treated LLINs (Permanet 3.0 and OlysetPlus) could be a productive strategy to replace conventional LLINs in the two targeted regions.

## Abbreviations

IRS: indoor residual spraying
USAID: United States agency for international development
WHO: world health organization
LLIN: long lasting insecticidal nets
NMCP: national malaria control program
MFO: mixed function oxidases
GST: glutathione S-transferase
PBO: piperonyl butoxide
